# Ultrasound Probe and Hand-Eye Calibrations for Robot-Assisted Needle Biopsy

**DOI:** 10.3390/s22239465

**Published:** 2022-12-03

**Authors:** Jihao Liu, Wenyuan Sun, Yuyun Zhao, Guoyan Zheng

**Affiliations:** Institute of Medical Robotics, School of Medical Engineering, Shanghai Jiao Tong University, Shanghai 200240, China

**Keywords:** robot-assisted, ultrasound guided, biopsy, hand-eye, calibration

## Abstract

In robot-assisted ultrasound-guided needle biopsy, it is essential to conduct calibration of the ultrasound probe and to perform hand-eye calibration of the robot in order to establish a link between intra-operatively acquired ultrasound images and robot-assisted needle insertion. Based on a high-precision optical tracking system, novel methods for ultrasound probe and robot hand-eye calibration are proposed. Specifically, we first fix optically trackable markers to the ultrasound probe and to the robot, respectively. We then design a five-wire phantom to calibrate the ultrasound probe. Finally, an effective method taking advantage of steady movement of the robot but without an additional calibration frame or the need to solve the AX=XB equation is proposed for hand-eye calibration. After calibrations, our system allows for in situ definition of target lesions and aiming trajectories from intra-operatively acquired ultrasound images in order to align the robot for precise needle biopsy. Comprehensive experiments were conducted to evaluate accuracy of different components of our system as well as the overall system accuracy. Experiment results demonstrated the efficacy of the proposed methods.

## 1. Introduction

Needle biopsy is a well-established procedure that allows for examination of abnormal tissue within the body. For example, percutaneous needle biopsy of suspected primary bone neoplasms is a well-established procedure in specialist centers [1]. Fine needle biopsy has long been established as an accurate and safe procedure for tissue diagnosis of breast mass [2,3]. Amniocentesis is a technique for withdrawing amniotic fluid from the uterine cavity using a needle [4,5,6,7]. Often, these procedures are performed under image guidance. Although some of the needle biopsy procedures can be guided using imaging modalities such as fluoroscopy, CT, MRI, single-photon emission computed tomography (SPECT), positron emission tomography (PET), and optical imaging, there are procedures such as amniocentesis which require continuous ultrasound (US) guidance when taking the safety of the mother and the baby into consideration. US is regarded as one of the most common imaging modalities for needle biopsy guidance as it is relatively cheap, readily available, and uses no ionizing radiation.

US-guided needle biopsies are often accomplished with hand held and stereotactic biopsy procedure, which are operator dependent. Moreover, such procedures require extensive training exercises, are difficult to regulate, and are more challenging to perform when small lesions are found. Consequently, hand held US-guided biopsies do not always yield ideal results.

To address these challenges, one of the proposed technologies is to integrate a robotic system with US imaging [3,8]. In such a robot-assisted, US-guided needle biopsy system, it is essential to conduct calibration of a US probe and to perform hand-eye calibration of the robot in order to establish a link between intra-operatively acquired US images and robot-assisted needle insertion. Based on a high-precision optical tracking system, novel methods for US probe and robot hand-eye calibration are proposed. Specifically, we first fix optically trackable markers to the US probe and to the robot, respectively. We then design a five-wire phantom to calibrate the US probe. Finally, an effective method taking advantage of steady movement of the robot but without the need to solve the AX=XB equation is proposed for hand-eye calibration. After calibration, our system allows for in situ definition of target lesions and aiming trajectories from intra-operatively acquired US images in order to align the robot for precise needle biopsy. The contributions of our paper can be summarized as:

- We design a five-wire phantom. Based on this phantom, we propose a novel method for ultrasound probe calibration.
- We propose an effective method for hand-eye calibration, which unlike previous work, does not need to solve the AX=XB equation, or a calibration frame.
- Comprehensive experiments are conducted to evaluate the efficacy of the proposed calibration methods as well as the overall system accuracy.

### Related Work

Different robotic systems have been developed for US-guided procedures. The robot has to know the spatial information of the target lesion and the aiming trajectory from the US image in order to realize the needle biopsy. The performance of the needle biopsy is dependent upon the image-to-robot registration accuracy.

Rapid and accurate US probe calibration depends on a well-designed phantom, which is expected to reduce the operation time and to improve the accuracy level. There exist different types of calibration phantom [9]. When a point phantom or plane phantom is used, it is very difficult to align the scan probe with the targets [10,11]. Moreover, these methods rely on a manual segmentation that is time-consuming and labor-intensive. The N-wire phantom was designed to solve the alignment problem [12,13,14]. However, it heavily depends on the known geometry constraint [15], which cannot be precisely satisfied considering the errors in fiducial detections from US images. To address the problem, arbitrary wire phantoms were proposed [16,17].

Hand-eye calibration aims to determine the transformation between a vision system and a robot arm system. The hand-eye calibration methods are different due to various kinds of vision devices and various fixing locations [18]. Generally, an additional calibration frame is required for the hand-eye calibration to identify the extrinsic and intrinsic parameters of the camera [19,20]. Furthermore, it is addressed by solving the form of AX=XB that formulates the closed-loop system [21]. Different methods and solutions have been developed, including simultaneous closed-form solution [22], separable closed-form solutions [23], and iterative solutions [24]. The first autonomous hand-eye calibration was proposed by Bennett et al. [25] to identify all parameters of the internal models of both the camera and the robot arm system by an interactive identification method. There also exist methods to identify the hand-eye transformation by recognizing movement trajectories of the reference frame corresponding to fixed robot poses [26]. In such methods, it is critical to choose appropriate poses and movement trajectories in order to realize a rapid and reliable calibration.

## 2. Overview of Our Robot-Assisted Ultrasound-Guided Needle Biopsy System

Our robot-assisted US-guided needle biopsy system consists of a master computer equipped with a frame grabber (DVI2USB 3.0, Epiphan Systems Inc., Ottawa, ON, Canada), an US machine (ACUSON OXANA2, Siemens Healthcare GmbH, Marburg, Germany) with a 45-mm linear array probe of 9L4 Transducer (Siemens Medical Solutions USA Inc., Pennsylvania, CA, USA), an optical tracking camera (Polaris Vega XT, Northern Digital Inc., Ontario, ON, Canada), and a robot arm (UR 5e, Universal robots Inc., Odense, Denmark) with a biopsy guide. Via the frame grabber, the master computer can grab real-time US images with a frequency of 10 Hz. It also communicates with the tracking camera to get poses of different tracking frames and with the remote controller of the UR robot in order to realize a steady movement and to receive feedback information, such as robot poses.

**Figure 1:**
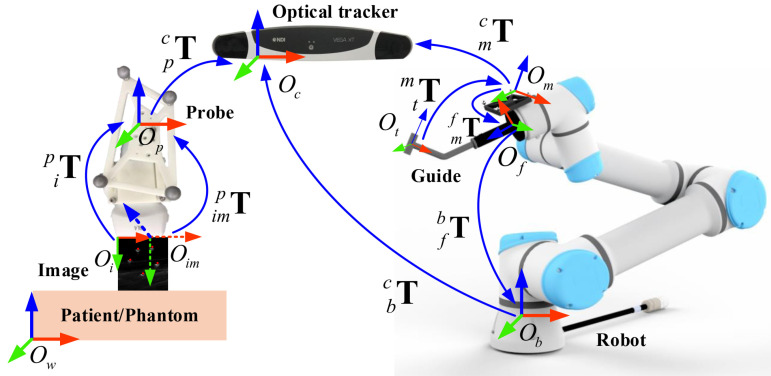
The involved coordinate systems in our robot-assisted US-guided biopsy system. During a needle biopsy procedure, the pose of the guide is adjusted to align with the biopsy trajectory planned in an acquired US image. See the main text for detailed descriptions.

During a needle biopsy procedure, the target lesion and the aiming trajectory are planned in the US image grabbed by the master computer. Then, the pose of the guide will be adjusted to align with the planned biopsy trajectory. Thus, it is essential to determine the spatial transformation from the two-dimensional (2D) US imaging space to the three-dimension (3D) robot space, as shown in Figure 1. The transformation can be obtained via three different calibration procedures, including US probe calibration, hand-eye calibration, and TCP (Tool Center Point) calibration.

A robot-assisted ultrasound-guided needle biopsy procedure involves following coordinate systems (COS) as shown in Figure 1. The 3D COS of the optical tracking camera is represented by $O_{c}$; the 3D COS of the reference frame on the end effector is by $O_{m}$; the 3D COS of the robotic flange is by $O_{f}$; the 3D COS of the guiding tube is by $O_{t}$; the 3D COS of the robot base is by $O_{b}$; the 2D COS of the US image is by $O_{i}$; the 3D COS of the plane where the US image is located is by $O_{im}$; the 3D COS of the reference frame attached to the US probe is by $O_{p}$; the 3D COS of the reference frame attached to the patient/phantom is by $O_{w}$. At any time, poses of different tracking frames with respect to the tracking camera such as ${}_{m}^{c}T$, ${}_{p}^{c}T$, ${}_{w}^{c}T$, are known. At the same time, the pose of the robotic flange with respect to the robot base ${}_{f}^{b}T$ is known. This transformation information can be retrieved from the API (Application Programming Interface) of the associated devices.

A biopsy trajectory can be defined from an intra-operatively acquired US image by a target point $p_{0}={\left(p_{x},p_{y},0\right)}^{T}$ and a unit vector $v_{0}={\left(v_{x},v_{y},0\right)}^{T}$ that indicates the direction of the trajectory. To simplify the derivation and expression, the planned trajectory in the image COS $O_{i}$ is written in a format of a 4×2 matrix, as:

(1) $${}^{i}\Psi =\left(\begin{array}{cc} p_{0} & v_{0}\\ 1 & 0 \end{array}\right)$$

The planned trajectory in the robot-base COS is presented by ${}^{b}\Psi$, which is obtained by the following chain of transformations:

(2) $${}^{b}\Psi ={}_{c}^{b}T\cdot {}_{p}^{c}T\cdot {}_{i}^{p}T\cdot {}^{i}\Psi$$

where ${}_{c}^{b}T$ represents the homogeneous transformation of the tracking camera COS $O_{c}$ relative to the robot-base COS $O_{b}$ and is determined by:

(3) $${}_{c}^{b}T={}_{f}^{b}T\cdot {}_{m}^{f}T\cdot {}_{c}^{m}T$$

where ${}_{f}^{b}T$ represents the homogeneous transformation of the flange COS relative to the robot-base COS, and ${}_{c}^{m}T$ is the inverse of ${}_{m}^{c}T$, which is the homogeneous transformation of the COS of the reference frame on the end effector relative to the tracking camera COS $O_{c}$.

Similar to the definition of the planned trajectory, pose of the center line of the guiding tube in the robot-base COS can be defined by ${}^{b}\Phi$, which is defined by two end points of the center line, $P_{1}$ and $P_{2}$:

(4) $${}^{b}\Phi =\left(\begin{array}{cc} P_{2} & \frac{P_{1}-P_{2}}{\left|\right|P_{1}-P_{2}\left|\right|}\\ 1 & 0 \end{array}\right)$$

To realize the robotic assistance for needle biopsy, the robot is controlled to provide a corresponding pose, so that the center axis of the guiding tube is aligned with the planned trajectory, which can be modeled as:

(5) $${}^{b}\Phi \equiv {}^{b}\Psi$$

The complete system requires knowing three spatial transformations, i.e., ${}_{i}^{p}T$, ${}_{m}^{f}T$ and ${}_{t}^{m}T$, of which ${}_{i}^{p}T$ is obtained by US probe calibration, ${}_{m}^{f}T$ is by hand-eye calibration, and ${}_{t}^{m}T$ is by TCP (Tool Center Point) calibration. The accuracy of the spatial calibrations will affect the biopsy accuracy. Below, we will present details about these three calibration procedures.

## 3. Calibration Methods

### 3.1. US Probe Calibration

${}_{i}^{p}T$ is used to transform a pixel in the 2D US imaging space $O_{i}$ to the 3D-COS $O_{p}$ of the reference frame attached to the US probe. This transformation matrix is determined by a calibration procedure as described below.

**Figure 2:**
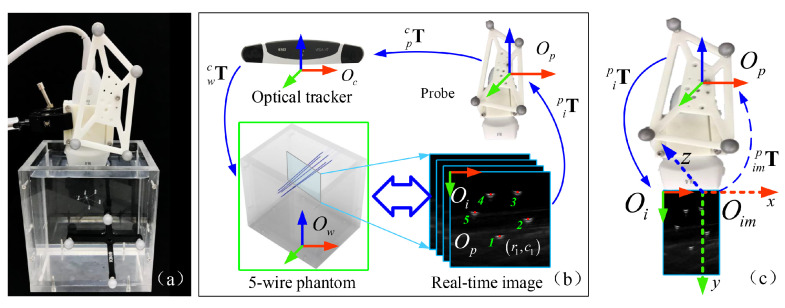
Schematic view of the US probe calibration based on a five-wire phantom; (**a**) experimental setup with a five-wire phantom; (**b**) spatial transformations involved in US probe calibration; (**c**) a schematic illustration on how to transform a pixel in the 2D US imaging space $O_{i}$ to the 3D-COS $O_{p}$ of the reference frame attached to the US probe.

To calibrate ${}_{i}^{p}T$, we design a five-wire phantom. The wire phantom uses five pieces of nylon wires with a diameter of 0.15 mm, as shown in Figure 2. These wires are designed not to be parallel to each other and are submersed in a water tank. During the US probe calibration process, we fix the scanning depth of the US to 5 cm, and the focus depth to 3.5 cm, which are selected based on typical clinical scenarios. The COS $O_{w}$ of the wire phantom is defined by fixing an optical reference frame to the phantom.

The transformation ${}_{i}^{p}T$ can be represented as:

(6) $${}_{i}^{p}T={}_{im}^{p}T\cdot {}_{i}^{im}T_{scale}$$

where the scaling matrix ${}_{i}^{im}T_{scale}$ describes the relationship between the local 2D US image COS $O_{i}$ and the 3D COS $O_{im}$, which defines the local COS of the plane where the US image is located (see Figure 2 for details); ${}_{im}^{p}T$ is the rigid body transformation between the 3D COS $O_{im}$ and the 3D COS $O_{p}$ of the reference frame attached to the US probe.

The scaling matrix ${}_{i}^{im}T_{scale}$ has the form:

(7) $${}_{i}^{im}T_{scale}=\left[\begin{array}{cccc} s_{x} & 0 & 0 & s_{tx}\\ 0 & s_{y} & 0 & 0\\ 0 & 0 & 1 & 0\\ 0 & 0 & 0 & 1 \end{array}\right]$$

where $s_{x}$ and $s_{y}$ represent the scaling parameters (mm/pixel) in the *x*- and *y*-direction, respectively; and $s_{tx}$ defines the translation between the origins of the local 2D US image COS $O_{i}$ and the 3D COS $O_{im}$. We can multiply ${}_{i}^{im}T_{scale}$ into ${}_{im}^{p}T$ to get ${}_{i}^{p}T$, which has the form:

(8) $${}_{i}^{p}T=\left(\begin{array}{cccc} {}_{i}^{p}r_{x} & {}_{i}^{p}r_{y} & {}_{i}^{p}r_{z} & {}_{i}^{p}t\\ 0 & 0 & 0 & 1 \end{array}\right)$$

where ${}_{i}^{p}r_{z}=\frac{{}_{i}^{p}r_{x}\times {}_{i}^{p}r_{y}}{\left|\right|{}_{i}^{p}r_{x}\times {}_{i}^{p}r_{y}\left|\right|}$, $s_{x}=\left|\right|{}_{i}^{p}r_{x}\left|\right|$, $s_{y}=\left|\right|{}_{i}^{p}r_{y}\left|\right|$, and ${}_{i}^{p}t$ is the sum of the translation components of matrices ${}_{i}^{im}T_{scale}$ and ${}_{im}^{p}T$. Thus, ${}_{i}^{p}T$ is determined by ${}_{i}^{p}r_{x}$, ${}_{i}^{p}r_{y}$ and ${}_{i}^{p}t$, which are all 3×1 vectors. Below, we present details on how to compute these three vectors.

The intersections between the US image plane and the wires are used to derive the transformation ${}_{i}^{p}T$. They are extracted from acquired US images by a semi-automatic point recognition algorithm [27]. Every detected intersection point is expressed as ${}^{i}P={\left(r,c,0,1\right)}^{T}$, where *r* and *c* indicate the location of a pixel at the *r*-th row and *c*-th column in the image. With ${}_{i}^{p}T$, the position of any intersection point can be transformed to the 3D COS $O_{p}$, as:

(9) $${}^{p}P={}_{i}^{p}T\cdot {}^{i}P$$

By simple mathematical operations, (9) can be rewritten as:

(10) $${}^{p}P=M_{a}\left(r,c\right)\cdot \alpha$$

where

(11) $$M_{a}\left(r,c\right)=\left(\begin{array}{cccccccccc} r & 0 & 0 & c & 0 & 0 & 1 & 0 & 0 & 0\\ 0 & r & 0 & 0 & c & 0 & 0 & 1 & 0 & 0\\ 0 & 0 & r & 0 & 0 & c & 0 & 0 & 1 & 0\\ 0 & 0 & 0 & 0 & 0 & 0 & 0 & 0 & 0 & 1 \end{array}\right)$$

(12) $$\alpha ={\left(\begin{array}{cccc} {}_{i}^{p}r_{x}^{T} & {}_{i}^{p}r_{y}^{T} & {}_{i}^{p}t^{T} & 1 \end{array}\right)}^{T}$$

The image-based points {${}^{p}P$} can be further transformed to the 3D COS $O_{w}$ via the transformations of the reference frame attached to the phantom ${}_{w}^{c}T$ and the reference frame attached to the US probe ${}_{p}^{c}T$ with respect to the tracking camera:

(13) $${}^{w}P={}_{c}^{w}T\cdot {}_{p}^{c}T\cdot {}^{p}P$$

The intersection point is on a straight wire which is rigidly attached to the phantom and can be modeled in the phantom COS $O_{w}$ as:

(14) $${}^{w}M\cdot {}^{w}P=0$$

where ${}^{w}M$ is a 2×4 coefficient matrix of a line equation in the phantom COS $O_{w}$, which can be determined if we know two points on the wire. This is done by digitizing the two end points of the wire using a tracked pointer. By combining (13) and (14), we have:

(15) $${}^{w}M\cdot {}_{c}^{w}T\cdot {}_{p}^{c}T\cdot {}^{p}P=0$$

The point recognition algorithm [27] will generate detection points with noise. To model such detection noise, we aim to compute the calibration parameters α by solving following optimization problem:

(16) $$\min:\sum_{i=1}^{k}\sum_{j=1}^{5}∥{}_{j}^{w}M\cdot {}_{c}^{w}T\cdot {}_{p}^{c}T\cdot M_{a}\left(r_{i,j},c_{i,j}\right)\cdot \alpha ∥$$

where $\left(r_{i,j},c_{i,j}\right)$ represents the location of the intersection pixel between the *j*-th wire with the *i*-th US image. ${}_{j}^{w}M$ is the corresponding known coefficient matrix of the *j*-th wire in the phantom COS $O_{w}$. After obtaining α, we can compute transformation ${}_{i}^{p}T$ according to (8) to finish the US probe calibration.

### 3.2. Hand-Eye Calibration

**Figure 3:**
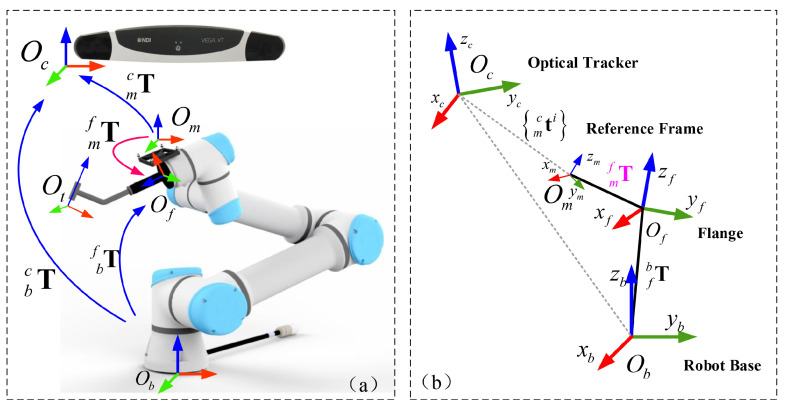
A schematic illustration of spatial transformations involved in the hand-eye calibration. (**a**) the set up; (**b**) the coordinate systems.

The hand-eye calibration is to establish the spatial transformation between the optical tracking camera and the robot. In this work, the hand-eye calibration is to derive the transformation ${}_{m}^{f}T$ of the optical reference frame attached to the end effector with respect to the robot base. Generally, the hand-eye transformation is represented by a homogeneous matrix ${}_{m}^{f}T$, which is composed of a rotation matrix ${}_{m}^{f}R$ and a translation vector ${}_{m}^{f}t$. Our hand-eye calibration procedure involves four 3D COSs as shown in Figure 3, including the robot-base COS $O_{b}$, the flange COS $O_{f}$, the tracking camera COS $O_{c}$, and the COS $O_{m}$ of reference frame attached to the end effector.

A conventional way to solve the hand-eye calibration problem requires solving the AX=XB equation. In this study, instead of solving the AX=XB equation, we propose a novel hand-eye calibration method that takes advantage of steady movement of the robot without an additional calibration frame. Specifically, we observe that the orientation of the reference frame changes only if the flange rotates. By controlling the flange to move in two different types of trajectories and by tracking the poses of the reference frame attached to the robot with respect to the tracking camera during the movement, we can compute the rotation matrix ${}_{m}^{f}R$ and the translation vector ${}_{m}^{f}t$ of the hand-eye calibration matrix, separately.

**Figure 4:**
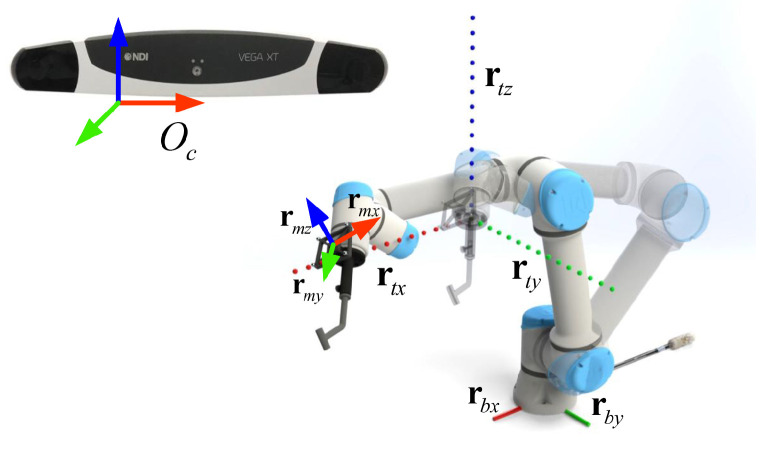
Movement trajectories of the reference frame for identifying the three column vectors, $r_{tx}$, $r_{ty}$, and $r_{tz}$, of the rotation matrix ${}_{b}^{c}\hat{R}$. During the movement, we keep the orientation of the flange unchanged under the observation of the tracking camera.

In the definition of the rotation matrix, the column vector of the rotation matrix indicates the components of coordinate axis of a COS relative to another COS. As shown in Figure 4, it is feasible to move the flange along the three coordinate axes of the robot-based COS $O_{b}$ while keeping the same orientation. Consequently, three line trajectories of the reference frame are recorded by the tracking camera which can be respectively used to compute the three column vectors of the rotation matrix ${}_{b}^{c}R$. In detail, we compute three unit vectors $r_{tx}$, $r_{ty}$ and $r_{tz}$ from the recorded trajectories, which represent the direction of the three coordinate axes of $O_{b}$ in the tracking camera COS $O_{c}$.

Hence, the rotation matrix ${}_{b}^{c}\hat{R}$ can be written as:

(17) $${}_{b}^{c}\hat{R}=\left(r_{tx},r_{ty},r_{tz}\right)$$

Considering the potential tracking errors, we decompose (17) with singular value decomposition (SVD) to preserve the orthogonality. The result (17) is:

(18) $$\left\{\begin{array}{c} {}_{b}^{c}\hat{R}=\mathrm{US}V^{T}\\ {}_{b}^{c}R=sign\left(\det\left(S\right)\right)UV^{T} \end{array}\right.$$

where $\det\left(\cdot \right)$ indicates the matrix determinant, and $sign\left(\cdot \right)$ is the sign function.

Then, the rotation matrix ${}_{m}^{f}R$ can be obtained through a chain of spatial transformations:

(19) $${}_{m}^{f}R^{i}={}_{b}^{f}R^{i}\cdot {}_{c}^{b}R\cdot {}_{m}^{c}R^{i}$$

where the right subscript *i* indicates the *i*-th points in the movement trajectories. ${}_{b}^{f}R$ is the inverse of ${}_{f}^{b}R$. ${}_{m}^{c}R$ is the orientation matrix of the reference frame attached to the robot with respect to the tracking camera.

Following (19), each point on the trajectories will give a different ${}_{m}^{f}R^{i}$ when taking tracking errors into consideration. We define a 3×9 matrix ${}_{f}^{m}M^{i}$ by column vectors ${r_{mx}}^{i}$, ${r_{my}}^{i}$, and ${r_{mz}}^{i}$ of ${}_{m}^{f}R^{i}$, as well as a 9×1 column vector β by column vectors $r_{mx}$, $r_{my}$, and $r_{mz}$ of the rotation matrix ${}_{m}^{f}R$. Because a rotation matrix is orthogonal, we further optimize the hand-eye calibration by using a least-squares fitting, as:

(20) $$\min:\sum_{i=1}^{k}∥{}_{f}^{m}M^{i}\cdot \beta -b∥$$

where

(21) $$\left\{\begin{array}{c} {}_{f}^{m}M^{i}=\left(\begin{array}{ccc} {\left(r_{mx}^{i}\right)}^{T} & 0_{1\times 3} & 0_{1\times 3}\\ 0_{1\times 3} & {\left(r_{my}^{i}\right)}^{T} & 0_{1\times 3}\\ 0_{1\times 3} & 0_{1\times 3} & {\left(r_{mz}^{i}\right)}^{T} \end{array}\right)\\ \beta ={\left(r_{mx}^{T},r_{my}^{T},r_{mz}^{T}\right)}^{T}\\ b={\left(1,1,1\right)}^{T} \end{array}\right.$$

$0_{1\times 3}$ is a 1×3 zero vector.

We can then obtain the rotation matrix ${}_{m}^{f}R$ in terms of β, and preserve its orthogonality by using SVD.

After obtaining the rotation matrix ${}_{m}^{f}R$, we can compute the rotation matrix ${}_{b}^{c}R$ at any time point. Now, we need to compute the translation vector ${}_{m}^{f}t$, which represents the offset of the origin of the COS $O_{m}$ relative to the flange COS $O_{f}$. This is done by controlling the movement of the robot such that the flange is rotated around its origin and by maintaining a fixed relationship between the camera and the robot base during the movement. Then, considering two different poses indexed by *i* and *j* in the rotational trajectory, we have:

(22) $$\left\{\begin{array}{c} {}_{m}^{c}T^{i}={}_{b}^{c}T\cdot {}_{f}^{b}T^{i}\cdot {}_{m}^{f}T\\ {}_{m}^{c}T^{j}={}_{b}^{c}T\cdot {}_{f}^{b}T^{j}\cdot {}_{m}^{f}T \end{array}\right.$$

As we are only interested in the translational part, we can decompose all the homogeneous transformations according to the block operation of the matrix to obtain:

(23) $$\left\{\begin{array}{c} {}_{m}^{c}t^{i}={}_{b}^{c}R\cdot {}_{f}^{b}R^{i}\cdot {}_{m}^{f}t+{}_{b}^{c}R\cdot {}_{f}^{b}t+{}_{b}^{c}t\\ {}_{m}^{c}t^{j}={}_{b}^{c}R\cdot {}_{f}^{b}R^{j}\cdot {}_{m}^{f}t+{}_{b}^{c}R\cdot {}_{f}^{b}t+{}_{b}^{c}t \end{array}\right.$$

**Figure 5:**
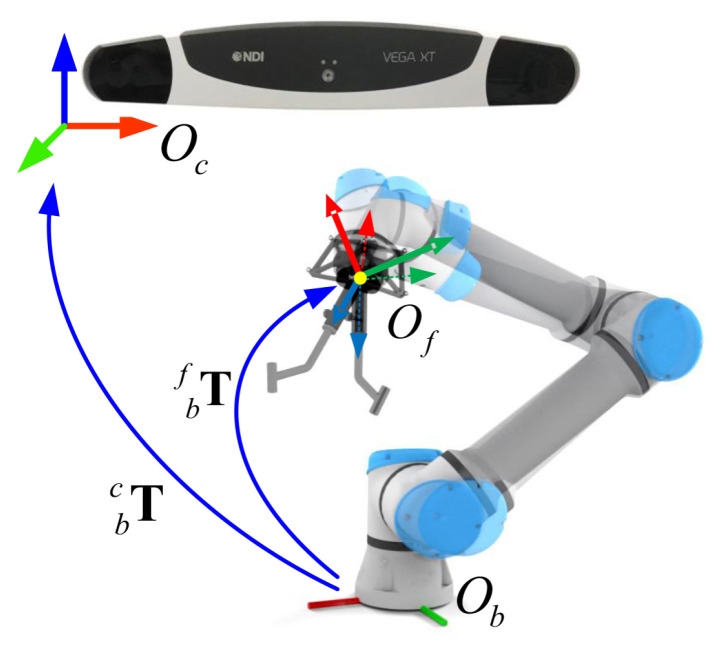
Rotating around the origin of the flange COS. The yellow point indicates the origin of the flange COS. During the rotation, we maintain a fixed relationship between the camera and the robot base.

In deriving above equations, as shown in Figure 5, we use the properties (1) that the flange is rotated around its origin, thus ${}_{f}^{b}t$ is constant and (2) that we maintain a fixed relationship between the camera and the robot base, thus ${}_{b}^{c}R$ and ${}_{b}^{c}t$ are constant. With a simple mathematical manipulation, we have:

(24) $${}_{m}^{c}t_{i}-{}_{m}^{c}t_{j}={}_{b}^{c}R\cdot \left({}_{f}^{b}R_{i}-{}_{f}^{b}R_{j}\right)\cdot {}_{m}^{f}t$$

In above equation, we would like to estimate ${}_{m}^{f}t$ while all other elements are either known or can be retrieved from the corresponding device’s API. Similarly, we can improve the translation vector calibration by using a least-squares fitting.

### 3.3. TCP Calibration

**Figure 6:**
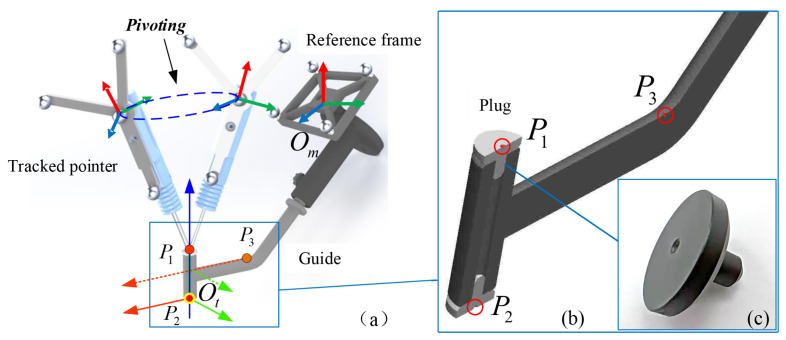
TCP Calibration by using a tracked pointer. (**a**) positions of points $P_{1}$, $P_{2}$ and $P_{3}$ are obtained by pivoting, which are used to build the local COS $O_{t}$ of the guiding tube. Specifically, the origin of he local COS $O_{t}$ is located at $P_{2}$. (**b**) Two points $P_{1}$ and $P_{2}$ are at the center of plugs inserting into the guiding tube, and the third point $P_{3}$ is on the guide. (**c**) A plug is designed for digitizing the end point of the guiding tube.

We need to conduct the TCP calibration in order to realize the closed-loop vision control on the pose of the guide under the tracking camera. The TCP calibration is a procedure to estimate the transformation ${}_{t}^{m}T$ of the COS $O_{t}$ defined on the guiding tube relative to the the COS $O_{m}$ of the reference frame attached to the end effector. In this calibration procedure, three COSs are utilized, including the tracking camera COS $O_{c}$, the local COS $O_{t}$ of the guiding tube, and the COS $O_{m}$, as shown in Figure 6.

As shown in Figure 6, the local COS $O_{t}$ of the guiding tube can be determined by three points, where $P_{1}$ and $P_{2}$ are two end points on the center axis of the guiding tube, and $P_{3}$ is a point on the guide. In order to determine two end points, plugs with a sharp indent are designed and inserted into the guiding tube. We then obtain the positions of these three points by using a tracked pointer pivoting at the corresponding indent.

In the local COS $O_{t}$, the origin is defined by the point $P_{2}$, the *z*-axis is determined by $P_{1}$ and $P_{2}$, and the *x*-*z* plane is the plane containing the three points. The coordinate axes can be modeled by the three points. $P_{1}$, $P_{2}$, and $P_{3}$ are all column vectors. We further obtain the homogeneous transformation ${}_{c}^{t}T$ by its origin and coordinate axes as:

(25) $${}_{t}^{c}T=\left(\begin{array}{cc} {}_{t}^{c}R & {}_{t}^{c}t\\ 0 & 1 \end{array}\right)=\left(\begin{array}{cccc} r_{x} & r_{y} & r_{z} & P_{2}\\ 0 & 0 & 0 & 1 \end{array}\right)$$

where

(26) $$\left\{\begin{array}{c} r_{x}=a_{13}\times a_{12}\times a_{12}\\ r_{y}=a_{13}\times a_{12}\\ r_{z}=a_{12} \end{array}\right.$$

(27) $$\left\{\begin{array}{c} a_{12}=\left(P_{1}-P_{2}\right)/∥P_{1}-P_{2}∥\;\\ a_{13}=\left(P_{1}-P_{3}\right)/∥P_{1}-P_{3}∥\; \end{array}\right.$$

We then combine ${}_{t}^{c}T$ with the pose ${}_{c}^{m}T$ of the reference frame to obtain the transformation ${}_{t}^{m}T$ as:

(28) $${}_{t}^{m}T={}_{c}^{m}T\cdot {}_{t}^{c}T$$

## 4. Evaluations and Experiments

### 4.1. Performance Evaluation

For the robot-assisted needle biopsy, the target point $p_{p}$ and vector $v_{p}$ of the trajectory direction are planned in an acquired US image. By the transformation ${}_{i}^{p}T$, they are transformed from the US imaging space into the physical space. Hence, the results of spatial calibrations affect the system performance. The US probe calibration affects the recognition and reconstruction on the planned trajectory, while the hand-eye calibration and the TCP calibration affects the accuracy of the robot control.

Accuracy evaluation on the US probe calibration is conducted by comparing reconstructed points, lines, and planes with the corresponding ground truth. With the aid of the tracking camera, the detected points in US images are reconstructed in the phantom space. The deviation between the recognized points and the digitized wire, which is used as the ground truth, and the incline angle between the fitted line and the digitized wire are used to evaluate the calibrations.

**Figure 7:**
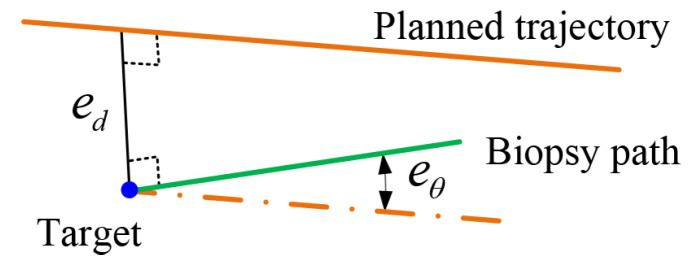
Metrics used to evaluate the accuracy including the angle $e_{\theta }$ as well as the distance $e_{d}$ between two spatial lines.

For the robotic system, the system performance is quantified by the deviations between the actual path $p_{a}$ and the planned biopsy trajectory. The deviations consist of the incline angle $e_{\theta }$ (unit: ${}^{\circ }$), as well as the distance $e_{d}$ (unit: mm) between the planned target point to the biopsy path, as shown in Figure 7.

### 4.2. Validation of the US Probe Calibration

**Figure 8:**
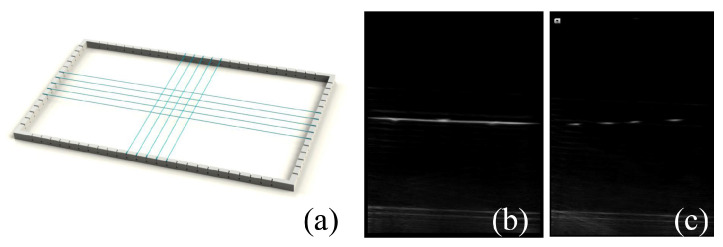
Validation wire phantom. (**a**) a supporting frame with crossing wires; (**b**) one of the US images intersecting with a transverse wire; (**c**) one of US images intersecting with longitudinal wires.

As shown in Figure 8, a plane-wire phantom was designed to verify the US probe calibration. Five longitudinal wires (LWs) and five transverse wires (TWs) were woven on a supporting frame, which was submerged in a water tank. The diameter of the wires was 0.15 mm. The span distance between the paralleled wires was about 10 mm. We used the semi-automatic point recognition algorithm [27] as we used in the probe calibration to recognize the intersection points between the US image plane and the validation wire phantom, which were represented as a set of pixels. We also established the line equations of the validation wire phantom using a tracked pointer, which was used as the ground truth.

### 4.3. Validation of Hand-Eye Calibration

**Figure 9:**
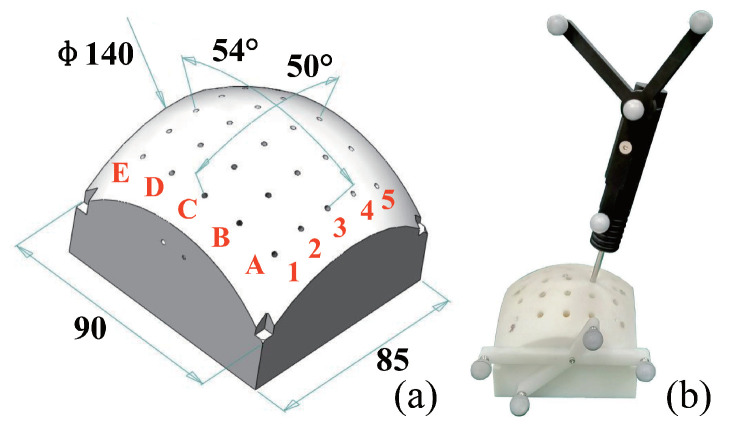
Plastic phantom for verifying hand-eye calibration. (**a**) geometric parameters; (**b**) using a tracked pointer to digitize actual drilling paths inside the phantom.

A plastic phantom fabricated by 3D printing was used for evaluating the validation of the hand-eye calibration and the TCP calibration. The phantom had a dimension of 140×90×85 mm ${}^{3}$. In addition, the phantom was designed with 5×5 drilling trajectories. As shown in Figure 9, the location of the drilling trajectories inside the plastic phantom was coded in alpha-numeric form. The robot was controlled to align a ϕ4 mm drilling bit with the planned trajectory. After drilling, a tracked pointer was used to digitize the drilled paths.

### 4.4. Blueberry Biopsy Experiments

**Figure 10:**
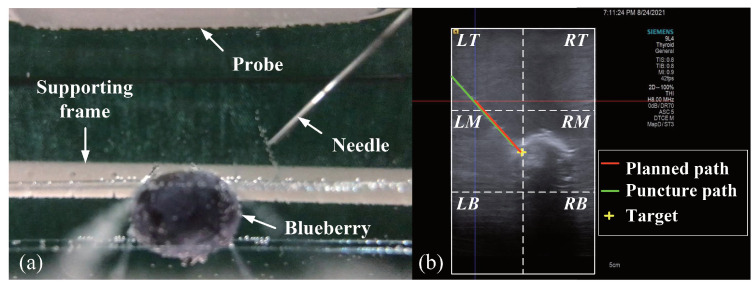
A schematic illustration of the setup for the blueberry biopsy experiments (**a**) and a schematic view of the partition of the water tank (**b**).

We designed biopsy experiments on a blueberry submerged in a water tank as shown in Figure 10. The target blueberry had a size of ϕ14.5 mm × 9.6 mm, and a biopsy needle had a diameter of 0.8 mm. We divided the water tank into 3×2 blocks and fixed the blueberry in the lower four blocks to simulate deep seated lesions. Moreover, the incline angle of the planned trajectory was varied over the range $30^{\circ }$ to $60^{\circ }$. The biopsy path can be real-time tracked by the ultrasound system. Thus, the biopsy accuracy was quantified by path deviations.

### 4.5. Tumor Phantom Biopsy Experiments

**Figure 11:**
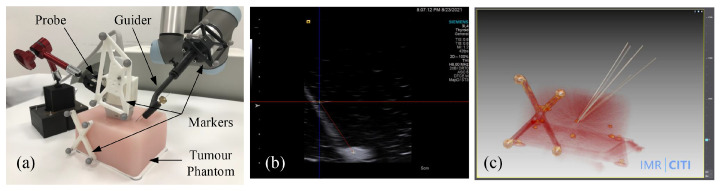
Soft tumor phantom biopsy experiments. (**a**) experimental setup including a robot arm with the guide, the soft tumor phantom with a reference frame atached, and the biopsy needle; (**b**) US image of a biopsy needle inserting into the tumor phantom; (**c**) CT image of the phantom after needle insertions.

We further conducted biopsy experiments on a soft tumor phantom (LYDMED, China) to validate the potential of the proposed system for tumor biopsy. The soft tumor phantom is made of silicon rubber and has a size of 150×120×80 mm ${}^{3}$, as shown in Figure 11.

There is a simulated tumor with a diameter of about 10 mm embedded inside the soft phantom. In addition, an optical reference frame was fixed to the phantom. The planned trajectories in the COS of the reference frame were obtained by using the method introduced in [28], which was treated as the ground truth. A needle with a diameter of 0.8 mm was inserted into the phantom via the passage of guide, and it was kept inside the phantom. We repeated the same procedure six times, and every time we planned different target points and aiming trajectories. After needle insertion, we obtained a CT scan of the phantom. The biopsy accuracy were then measured in the 3D CT imaging space.

## 5. Experimental Results

### 5.1. US Probe Calibration

During the US probe calibration, we acquired 110 frames of US images, of which 90 images were used as the data set to derive the transformation ${}_{i}^{p}T$, and the others were used as the test data to evaluate the calibration accuracy. We used the test set to reconstruct the five-wire phantom. The distance between the detected points and the adjacent wires, and the incline angle of the reconstructed lines, are presented in Table 1. An average incline angle of $0.3^{\circ }$ and an average distance of 0.85 mm were found.

### 5.2. Validation of US Probe Calibration

**Figure 12:**
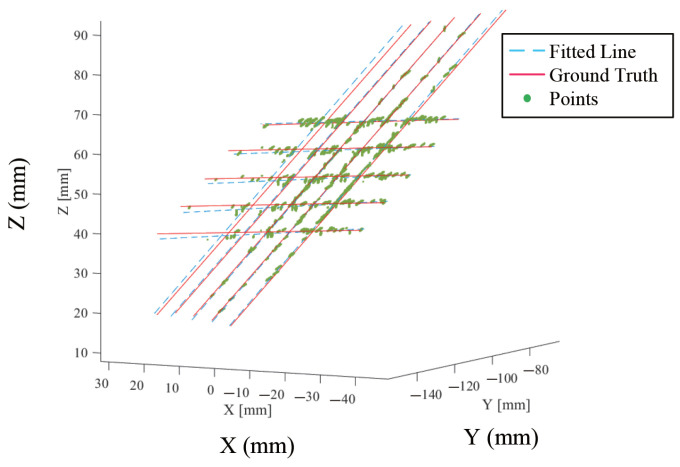
Validation of the US probe calibration. Red lines indicate the ground truth wires while blue lines are fitted lines. Green points are the points detected from the US images.

For the US probe calibration validation, 176 frames of images were acquired, and 26,868 intersection points were detected from these images, which were used to reconstruct the plane phantom, as shown in Figure 12. The incline angle of the normal vector of the fitted plane was $0.50^{\circ }$. The mean distance between the corresponding position of the detected points and the wires, and the mean incline angle between the fitted lines, are presented in Table 2. From this table, one can see that our US probe calibration method achieved sub-millimeter and sub-degree accuracy, which were accurate enough for our applications.

### 5.3. Validation of Hand-Eye Calibration

In the hand-eye calibration validation experiments, the distance and the incline angle between the drilling path and the planned trajectory are presented in Table 3. Specifically, we found that the mean distance deviation was 0.33 mm and the maximum distance deviation 0.67 mm. The mean and the maximum incline angle were $1.03^{\circ }$ and $2.44^{\circ }$, respectively. The relatively large angular error might be caused by the vibration of the guide during drilling.

### 5.4. Blueberry Biopsy Experiments

**Figure 13:**
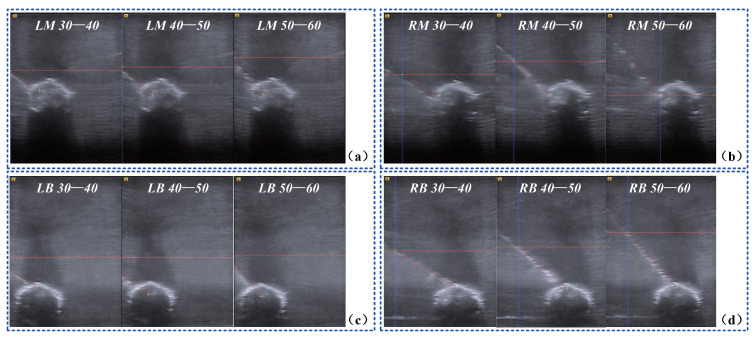
US images of the biopsy on a blueberry. (**a**) biopsy on the targets in the left middle block from three different angles; (**b**) biopsy on the targets in the right middle block from three different angles; (**c**) biopsy on the targets in the left bottom block from three different angles; (**d**) biopsy on the targets in the right bottom block from three different angles.

As shown in Figure 13, we quantified the deviations of the targets and the trajectories when the blueberry was submerged in different blocks of the water tank. The experimental results of the 72 times biopsy on a blueberry are presented in Table 4. An average distance error of 0.74 mm and an average angular error of $1.10^{\circ }$ were founded. Throughout the 72 times biopsy, the successful rate was 100%.

### 5.5. Tumor Phantom Biopsy Experiments

The overall system performance was evaluated by needle biopsy on a tumor phantom. Results of the tumor phantom experiment are presented in Table 5. The success rate of the needle biopsy into the tumor was 100%. An average distance error of 1.71 mm and an average angular error of $1.0^{\circ }$ were found. We attributed the relatively large errors to the elastic deformation of the biopsy needle during insertion. Nonetheless, the achieved accuracy is good enough for the target applications and is better than the results achieved by most of the state-of-the-art methods [2,3,8,13,29].

### 5.6. Comparison with the State-of-the-Art (SOTA) Methods

For US probe calibration, we compared the reconstruction accuracy with SOTA methods using other types of phantoms, including the method introduced by Wen et al. [15], the method based on an eight-wire phantom [17], the method based on an N-wire phantom [13], the method based on a pyramid phantom [14], and the method based on a Z-wire phantom [9]. In terms of the mean reconstruction accuracy, our method achieved the best result. Table 6 shows the comparison results.

Additionally, we also compared our method with other SOTA biopsy methods, including the method introduced by Tanaiutchawoot et al. [30], the method introduced by Treepong et al. [29], and the method introduced by Chevrie et al. [31]. Table 7 shows the comparison results, where the exact type of phantom, the achieved accuracy and the biopsy successful rate of each method are presented. From this table, one can see that our method achieved the best result in terms of both the accuracy and the biopsy successful rate.

## 6. Discussion

Previous studies of needle biopsy have emphasized the applications of fluoroscopy and CT as imaging modalities [32,33]. Compared with these imaging modalities, US has a major advantage in that it is free of risk from ionizing radiation to both the patient and staff. In addition, robot systems have the advantage to ensure the stability and accuracy [30,34]. Taking advantage of an ultrasound system and a robot arm, we developed and validated a robot-assisted system for a safe needle biopsy.

Three spatial calibration methods, including US probe calibration, hand-eye calibration, and TCP calibration, were developed for the robot-assisted biopsy system to realize a rapid registration of patient-image-robot. We validated the US probe calibration by reconstruction analysis of wire phantoms. Our method also achieved a higher accuracy than previously reported results [13,15,16,35]. Different from previous works [10,12,17], our US probe calibration is not dependent upon the known geometric parameters, which makes it easier to manufacture a calibration phantom. We further investigated a combination of the hand-eye calibration and TCP calibration by drilling experiments.

It is worth discussing the proposed hand-eye calibration method. Our method does not need to solve the equation “AX=XB” as required by previously introduced hand-eye calibration methods [36]. In comparison with methods depending on iterative solutions [24,25] or probabilistic models [22,37], our method is much faster. Our method also eliminates the requirement of an additional calibration frame as in [19,20]. Our hand-eye calibration transformation is derived based on the movement trajectories of the reference frame attached to the end effector, taking advantage of the steady movement of a robot.

There are limitations in our study. First, we did not consider the influence of respiratory motion, which may degrade the performance of the proposed system. Second, the accuracy of the proposed system was affected by the elastic deformation and friction of the target object, which conformed with the finding reported in [31]. Nonetheless, results from our comprehensive experiments demonstrated that the proposed robot-assisted system could achieve sub-millimeter accuracy.

## 7. Conclusions

In this paper, we developed a robot-assisted system for an ultrasound-guided needle biopsy. Specifically, based on a high-precision optical tracking system, we proposed novel methods for US probe calibration as well as for robot hand-eye calibration. Our US probe calibration method was based on a five-wire phantom and achieved sub-millimeter and sub-degree calibration accuracy. We additionally proposed an effective method for robot hand-eye calibration taking advantage of steady movement of the robot but without the need to solve the AX=XB equation. We conducted comprehensive experiments to evaluate the efficiency of different calibration methods as well as to evaluate the overall system accuracy. Results from our comprehensive experiments demonstrate that the proposed robot-assisted system has a great potential in various clinical applications.

## Figures and Tables

**Table 1:** Results of the US probe calibration.

	$e_{d}$ (mm)			$e_{\theta }\;{(}^{\circ })$
	Max.	Min.	Mean	
1	0.52	0.01	0.19	0.54
2	0.63	0.04	0.30	0.84
3	0.54	0.03	0.30	1.10
4	0.71	0.05	0.39	1.15
5	0.66	0.06	0.31	0.61
Average			0.30	0.85

**Table 2:** Results of the experiments on validation of US probe calibration.

	TWs		LWs	
	$e_{d}$ (mm)	$e_{\theta }\;{(}^{\circ })$	$e_{d}$ (mm)	$e_{\theta }\;{(}^{\circ })$
1	0.59	0.49	0.54	0.45
2	0.58	0.74	0.63	0.58
3	0.54	0.78	0.72	0.18
4	0.55	1.27	0.69	0.60
5	0.55	1.09	0.82	0.29
Average	0.56	0.87	0.68	0.42

**Table 3:** Results of the experiments on validation of hand-eye calibration.

	$e_{d}$ (mm), $e_{\theta }\;{(}^{\circ })$				
	A	B	C	D	E
1	/	0.17, 1.54	0.43, 1.16	0.46, 0.92	/
2	0.26, 1.19	0.28, 1.60	0.15, 0.83	0.37, 0.78	0.24, 0.86
3	0.20, 1.49	0.13, 0.63	0.44, 1.49	0.04, 0.81	0.60, 0.72
4	0.30, 1.04	0.47, 0.54	0.47, 1.17	0.67, 0.11	0.32, 0.98
5	/	0.01, 2.44	0.56, 1.17	0.35, 0.23	/

**Table 4:** Results of the blueberry biopsy experiments.

	Biopsy Angle (${}^{\circ }$)					
	30–40		40–50		50–60	
	$e_{d}$ (mm)	$e_{\theta }{(}^{\circ })$	$e_{d}$ (mm)	$e_{\theta }{(}^{\circ })$	$e_{d}$ (mm)	$e_{\theta }{(}^{\circ })$
LM	1.36	0.64	1.02	1.12	1.01	1.20
RM	0.58	0.36	0.05	0.41	0.83	0.48
LB	0.37	1.64	1.20	2.40	0.66	1.49
RB	0.42	0.03	1.11	2.54	0.33	0.35

**Table 5:** Results of the tumor phantom biopsy experiments.

	$e_{d}$ (mm)	$e_{\theta }{(}^{\circ })$
1	1.90	0.35
2	1.57	0.96
3	1.89	0.73
4	1.87	0.67
5	1.55	1.01
6	1.49	2.26
Average	1.71	1.00

**Table 6:** Comparison with other SOTA US probe calibration methods.

Method	Phantom Type	Mean Accuracy
Wen et al. [15]	Combined phantom and stylus	0.71 mm
Ahmad et al. [17]	Eight-wire phantom	1.67 mm
Carbaja et al. [13]	N-wire phantom	1.18 mm
Lindseth et al. [14]	Pyramid phantom	0.80 mm
Hsu et al. [9]	Z-wire phantom	0.70 mm
Ours	Five-wire phantom	0.62 mm

**Table 7:** Comparison with other SOTA biopsy methods. “-” indicates that the corresponding data are not available.

Method	Object	Mean Accuracy	Biopsy Successful Rate
Tanaiutchawoot et al. [30]	Soft phantom	3.44 mm	92 %
Treepong et al. [29]	Soft phantom	2.85 mm	80 %
Chevrie et al. [31]	Gelatin phantom	2.5 mm	-
Ours	Blueberry	0.74 mm	100 %
Ours	Soft phantom	1.71 mm	100 %

## Data Availability

Not applicable.

## Abbreviations

The following abbreviations are used in this manuscript:

2D	Two-dimension
3D	Three-dimension
API	Application Programming Interface
COS	Coordinate system
LB	Left bottom
LM	Left middle
LW	Longitudinal wire
RB	Right bottom
RM	Right middle
SVD	Singular value decomposition
TCP	Tool center point
TW	Transverse wire
US	Ultrasound
